# A Natural Plant Source-Tea Polyphenols, a Potential Drug for Improving Immunity and Combating Virus

**DOI:** 10.3390/nu14030550

**Published:** 2022-01-27

**Authors:** Mengyu Hong, Lu Cheng, Yanan Liu, Zufang Wu, Peng Zhang, Xin Zhang

**Affiliations:** 1 Department of Food Science and Engineering, Ningbo University, Ningbo 315211, China; a13056961758@163.com (M.H.); liuyanan@nbu.edu.cn (Y.L.); zxdqqzyc@163.com (Z.W.); 2 Department of Food Science, Rutgers University, New Brunswick, NJ 08901, USA; lc894@scarletmail.rutgers.edu; 3 Department of Student Affairs, Xinyang Normal University, Xinyang 464000, China

**Keywords:** tea polyphenols, COVID-19, gut-lung axis, immunity, antiviral effect

## Abstract

The coronavirus disease 2019 (COVID-19) is still in a global epidemic, which has profoundly affected people’s lives. Tea polyphenols (TP) has been reported to enhance the immunity of the body to COVID-19 and other viral infectious diseases. The inhibitory effect of TP on COVID-19 may be achieved through a series of mechanisms, including the inhibition of multiple viral targets, the blocking of cellular receptors, and the activation of transcription factors. Emerging evidence shows gastrointestinal tract is closely related to respiratory tract, therefore, the relationship between the state of the gut–lung axis microflora and immune homeostasis of the host needs further research. This article summarized that TP can improve the disorder of flora, reduce the occurrence of cytokine storm, improve immunity, and prevent COVID-19 infection. TP may be regarded as a potential and valuable source for the design of new antiviral drugs with high efficiency and low toxicity.

## 1. Introduction

Since the discovery of patients with coronavirus disease 2019 (COVID-19) in December 2019, the severe acute respiratory syndrome coronavirus 2 (SARS-CoV-2) epidemic has spread to 141 countries. As of 30 December 2021, there are more than 250 million confirmed cases worldwide and more than 5 million deaths [1]. The SARS-CoV-2 strains continue to mutate; as of now, the World Health Organization has named 11 SARS-CoV-2 strain variants. The spread of SARS-CoV-2 in almost all countries in the world is causing serious public health incidents, and social and economic turmoil. In the absence of specific effective treatment, the development of a vaccine has become the most urgent and important measure in the field of public health. The world has been racing to develop a safe and effective SARS-CoV-2 vaccine. Due to the limited speed of vaccination, it will take several more years to achieve complete herd immunity.

Like other RNA viruses, the replication of SARS-CoV-2 also requires RNA-dependent RNA polymerase (RdRp), which plays an important role in virus replication and transcription [2]. Coronavirus can be divided into four genera: α, β, γ, and δ. A total of seven coronaviruses that can infect humans have been found [3]. SARS-CoV-2, Middle East respiratory syndrome (MERS), and SARS-CoV belong to β-coronaviruses and can cause severe infections [4]. The remaining four coronaviruses cause only mild symptoms similar to the common cold in humans. Among them, human coronavirus (HCoV)-229E and HCoV-NL63 belong to α-coronaviruses. HCoV-OC43 and HCoV-HKU1 belong to β-coronaviruses [5]. The current SARS-CoV-2 virus has a close genomic similarity with SARS-CoV and MERS-CoV. It is likely that SARS-CoV-2 has a similar mechanism to evade cellular immune response. Escaping human cellular immunity is a common feature of the SARS-CoV-2. Complexity of the global vaccination plan has greatly increased, due to that mutated SARS-CoV-2 escapes the immune system [6]. Early studies have shown that down-regulation of type I interferon (IFN) is one of the strategies for coronaviruses to evade the immune response [7]. Recent research results confirmed that the secretion of type I IFN in critically ill patients with COVID-19 is impaired and the virus clearance rate is low [8].

Plant polyphenols from natural sources are popular in preventing many diseases including viral diseases [9]. Recently, biologically active molecules, including a polymeric polyphenol extracted from tea tree (*Camellia sinensis* L.) have been found to act as an effective SARS-CoV-2 main protease inhibitor [10]. The antiviral effect of polyphenols is due to the interaction between benzene ring and viral protein and/or RNA, or through their ability to interfere with host cell defense by regulating mitogen-activated protein kinase signal [11]. Tea polyphenols (TP) can inhibit the migration of neutrophils through the endothelial cell monolayer, thereby reducing vascular permeability [12]. It can also reduce neutrophil elastase, a proteolytic molecule involved in the body’s inflammatory response, which is associated with increased alveolar epithelial permeability [13]. Neutrophils lose the integrity of the endothelial cell barrier and produce reactive oxygen species, leading to imbalance in homeostasis, which may explain the cause of acute respiratory distress syndrome (ARDS) in patients with COVID-19. It is manifested by damage to the alveolar epithelial barrier and increased pulmonary microvascular permeability, causing severe respiratory disorders [14].

Although COVID-19 mainly damages the lungs, it also affects other parts of the body (such as the intestines) [15]. More and more evidence shows that there is a close relationship between the gastrointestinal tract and the respiratory tract. Microbes in the lungs do not seem to be the only cause of pneumonia. Microbes in the gut also seem to have some effect on lung health [16]. The homeostasis of the gut-lung axis affects the immune system and is helpful to resist COVID-19. Immune disorders participate in and even dominate many diseases; eating TP can relatively increase the number of probiotics and probiotics can shape intestinal microflora, significantly regulating the immunity of the host [17,18].

In this review, we discussed the interaction mechanism between TP, the main component of natural plant tea, and different targets of coronaviruses, including the existing SARS-CoV-2. TP may exert its effective function to treat COVID-19 from the perspective of the gut–lung axis. The treatment of coronavirus should not only start from suppressing the virus itself, but also improve the body’s immune system and reduce inflammation.

## 2. Pathogenesis and Symptoms of COVID-19 in Human

The pathogenesis of severely ill patients infected with SARS-CoV-2 is very complicated. After the infected people exhale the droplets carrying the virus and is inhaled by others, SARS-CoV-2 enters the nasal cavity and throat through this way to start a new round of infection. The key to virus selection of the nasal cavity is angiotensin-converting enzyme 2 (ACE2), which infects cells with ACE2 as the receptor and enters the cells of the body [19]. Subsequently, the infection spreads to the upper respiratory tract and then to the alveoli, where it targets type II lung cells and leads to ARDS and even death [20]. Finally, a large-scale systemic multi-organ infection may occur because the target of the virus is to attack the alveolar epithelial cells and vascular endothelial cells [21]. The infection and destruction of the tissue barrier cells allow the virus to enter the blood and lymphatic system, spreading to several organs including the heart [22], kidneys [23], liver, and brain [24].

**Figure 1:**
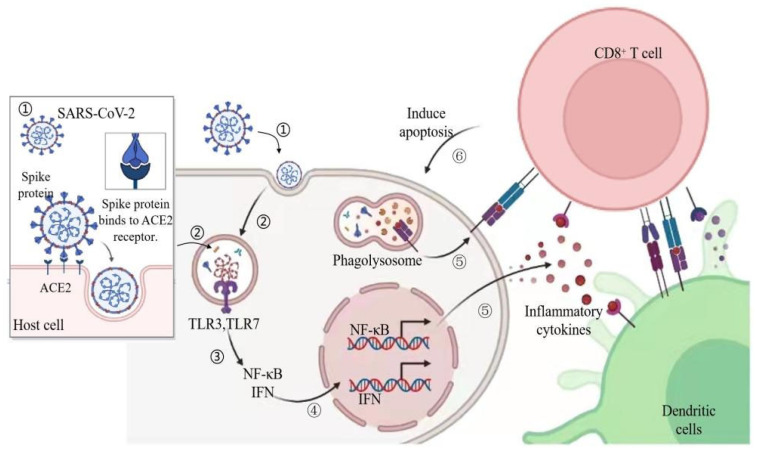
Immune response triggered by the SARS-CoV-2 entering the body. SARS-CoV-2 = severe acute respiratory syndrome coronavirus 2; ACE2 = angiotensin-converting enzyme 2; TLR = Toll-like receptor; IFN = interferon. Figure generated with BioRender (Toronto, ON, Canada).

ACE2-expressing cells distributed on the surface of the nose and lung mucosa can promote respiratory infections. However, ACE2 can also be expressed in cells of many other tissues, including endothelium, heart, intestine, and kidney. Therefore, these organs are also vulnerable to virus attacks. The significant sign of severe SARS-CoV-2 is cytokine release syndrome. SARS-CoV-2 respiratory tract infection epithelial cells activate monocytes, macrophages, and dendritic cells, leading to the secretion of a series of pro-inflammatory cytokines, including interleukin (IL)-6. IL-6 promotes the differentiation of T helper cell 17 and other lymphocyte changes. Human ACE2 is an entry receptor for SARS-CoV, SARS-CoV-2, and other human coronaviruses. The glycoprotein on the surface of the virus binds to the cell surface receptor ACE2, which plays an important modulatory role in the body’s renin-angiotensin-aldosterone system [25]. Viral RNA activates Toll-like receptor (TLR) 3 and 7. These receptors activate interferon regulatory factors and nuclear factor (NF)-κB signaling to induce inflammatory cytokines, including IFN. Dendritic cells collect antigens and migrate to lymphoid organs to induce adaptive immunity. CD8^+^ T cells induce apoptosis after recognizing antigens on dendritic cells or infected cells [26] (Figure 1). The physiological role of ACE2 is to regulate blood pressure. ACE2 transforms harmful vasoconstrictor angiotensin II to protective angiotensin 1–7, which are vasodilators [27]. In the autopsy of patients who died of ARDS, only high levels of pro-inflammatory cytokines were detected in ACE2^+^ cells infected with SARS-CoV [28]. The expression level of proinflammatory cytokines in ACE2 cells of patients who died of SARS-CoV was higher [28]. After virus infection, the immune system is activated and a variety of cytokines, including IFN, are induced. These cytokines promote the transcription and expression of ACE2 through their receptors activating downstream signal pathways. High expression of ACE2 can promote its infection, while low expression and non-expression can inhibit virus infection and even lead to infection failure [29]. Collectively, the invasion of ACE2 by SARS-CoV-2 may trigger inflammatory signals (Figure 1).

**Figure 2:**
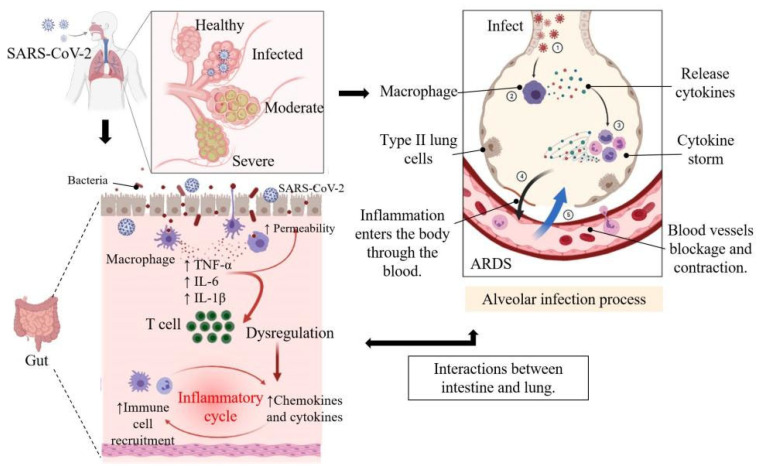
Reactions in the lung and intestine in patients infected with COVID-19. ARDS = acute respiratory distress syndrome. Figure generated with BioRender.

Studies have shown that in COVID-19 pneumonia, oxygen uptake impairment is partially caused by blockage or contraction of blood vessels in the lungs, not just the accumulation of alveolar edema fluid and alveolar congestion. Oxygen uptake disorders caused by the accumulation of alveolar edema fluid and alveolar congestion are common in pneumonia caused by other respiratory virus infections [30]. It is becoming increasingly clear that a series of complications induced by SARS-CoV-2 infection can damage multiple organs and tissues, from the kidneys to the brain [30] (Figure 2).

The clinical symptoms caused by SARS-CoV-2 in different populations vary greatly. Some infected people have no symptoms from beginning to end, and some infections show very serious clinical symptoms, and even lose their lives due to respiratory failure. Eighty percent of SARS-CoV-2 infected patients are mild patients. The symptoms of these patients may include fever, cough, sore throat, loss of smell, headaches, and muscle aches. The disease can be divided into three stages: pre-symptomatic, symptomatic, and the recovery period. Virus replication activates the body’s natural immune response, including the activation of macrophages, natural killer cells, dendritic cells, and a series of immune cells that secrete pro-inflammatory cytokines. In critically ill patients, natural immunity is over-activated and maintained at a high level for a long time, causing immune damage to tissues. In COVID-19 patients with mild to moderate symptoms, the cellular immune response of antigen-specific B and T cells can be detected within the first week after the onset of symptoms [31]. These patients have reduced B and T lymphocytes in the peripheral blood, which indicates a reduction in immune response and antibody production. In addition to lymphopenia, patients with new coronary pneumonia will also experience hypoproteinemia and neutropenia. These manifestations may be accompanied by multiple organ complications, which can lead to systemic organ failure, thrombus, mental and neurological complications, loss of sense of smell and taste, heart failure, acute kidney injury, diarrhea, and other gastrointestinal inflammation, and even death [17,32,33]. These complications are related to cytokine storm. Lung immune cells are over-activated to produce a large number of inflammatory factors, which form an inflammatory storm through the mechanism of positive feedback cycle. Once a cytokine storm is formed, the immune system will not only kill the virus, but also kill a large number of normal cells in the lungs, seriously damaging the ventilation function of the lungs, resulting in ARDS [34].

Worldwide, the establishment of a universal immune barrier is the most effective means to defeat the coronavirus epidemic. At present, the research and development of anti-SARS-CoV-2 vaccines and specific drugs should be accelerated.

## 3. Antioxidant, Anti-Inflammatory, and Anti-Viral Activity of Natural Polyphenols in Tea

Generally, β-coronaviruses produce about 800 kDa polypeptides during genome transcription. These polypeptides are hydrolyzed by papain-like protease (PL^pro^) and 3-chymotrypsin-like protease (3CL^pro^) to produce various proteins [35]. As 3CL^pro^ is necessary for SARS-CoV-2 maturation, it is urgent to find potential 3CL^pro^ inhibitors to develop antiviral drugs for SARS-CoV-2. Increasingly, studies are revealing that phyto-polyphenols play a vital part in the stoppage of coronavirus [36]. Many countries have put forward the concept of functional food, which can enhance the immune response and physiological function in the diet without taking medicines and injections. Under the current raging situation of COVID-19, maintaining a healthy and balanced immune system is important for the human body. Due to the high biological activities, especially antioxidant, anti-inflammatory, antibacterial, and anti-viral capabilities, plant polyphenols have attracted more attentions. Epigallocatechin gallate (EGCG) is the main component of TP. In the mouse model, it has been found that the daily non-toxic dose of EGCG (lower than 30 mg/kg, i.p. or 300 mg/kg, i.g.) can improve oxidative stress, cytokine storm, sepsis, and pulmonary fibrosis [37]. If these signs can be shown in humans, they will help to prevent or alleviate COVID-19 and the related symptoms.

### 3.1. Antioxidant, Anti-Inflammatory and Anti-Viral Activities of TP

Coronavirus causes a strong immune response in the body, which is regulated by closely related oxidative stress and inflammatory processes [18]. As oxidative stress plays an important role in lung injury, oxidative stress can induce apoptosis of alveolar epithelial cells, antioxidants have become a new treatment method [38]. Due to its chemical structure containing multiple hydroxyl groups, TP has high antioxidant activity. Natural polyphenol products with antioxidant and anti-inflammatory properties can be used as potential supplements to strengthen the immune and antioxidant defense system [39]. TP exerts its anti-inflammatory mechanism by regulating immune function and cytokine expression. In addition, TP reduces the production of reactive oxygen species (ROS) by promoting the expression of heme oxygenase 1, exert its antioxidant activity and upregulate endogenous antioxidant enzymes, such as superoxide dismutase and catalase and glutathione peroxidase [40]. A large amount of evidence shows that the increase of ROS and the loss of antioxidant defense mechanisms enhance the incidence of SARS-CoV infection, which is inseparable in the progression of respiratory diseases [41]. Due to age-related reduction of endogenous antioxidants and high levels of pro-inflammatory reactions, the risk of death from SARS-CoV-2 in the elderly is increased [42]. In the case of COVID-19 infection, systemic inflammatory response will stimulate the body to be in a state of stress. Oxidative stress is usually characterized by the activation of innate immunity and transcription factors (such as NF-κB), resulting in a cytokine storm in the host [18]. This association increases the morbidity and mortality of respiratory virus infections.

Further studies showed that different TP monomers or their derivatives have different inhibitory effects on influenza viruses, and even the same TP monomer has different inhibitory effects on different subtypes of the same influenza virus. The difference in antiviral activity can be attributed to the number and structure of the hydroxyl groups of the benzene ring, the presence of gallic acid groups, pyrogallol groups, and the EGCG skeleton itself also plays an important role [43]. For example, the inhibitory effect of EGCG on the hemagglutinin of H5N1 influenza virus is greater than that of epigallocatechin (EGC) [44], while the activity of EGC against H1N1 influenza virus is greater than that of EGCG [45]. Direct treatment of porcine reproductive and respiratory syndrome virus with EGCG seems to be more effective in suppressing viral infection, because EGCG at a concentration of 125 µM completely eliminates the infectivity of the virus [46]. Studies have shown that TP or its monomer components can resist human immunodeficiency virus infection in different ways [47]. EGCG can block the initial step of hepatitis C virus invasion by inhibiting the fusion of hepatitis C virus envelope and cell membrane and the transmission of virus between cells [48]. It can be seen from the inhibitory effect of EGCG in various viruses that EGCG is a broad-spectrum antiviral substance, and its mechanism of action varies with the type of IFN. Starting from the known antiviral effect of TP, it is conceived whether TP can also effectively inhibit the replication of SARS-CoV-2. Recent studies have also confirmed that TP can inhibit SARS-CoV-2 in vitro, so TP is expected to become an anti-novel coronavirus drug or supplement [36,49].

During viral infections where disrupted redox balance and aggravated inflammatory storm, it is predicted that supplementing with TP antioxidants may counteract oxidative stress, enhance the antioxidant, anti-inflammatory, and anti-viral capabilities of cells, thereby improving health.

### 3.2. TP Inhibits the 3CL^pro^ Activity of SARS-CoV-2

**Figure 3:**
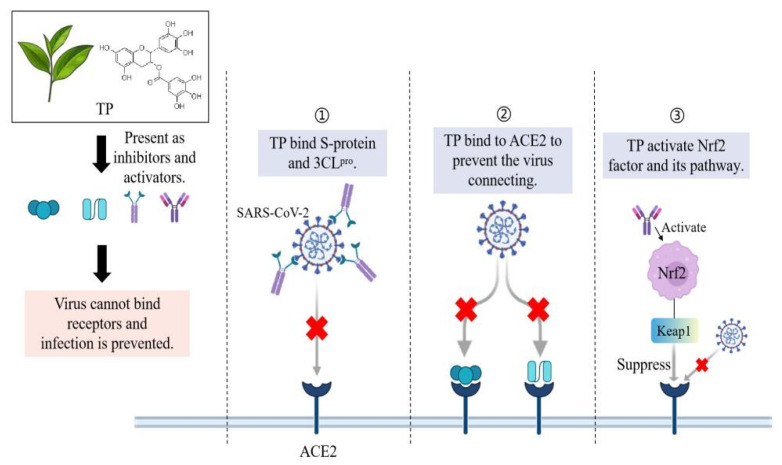
Tea polyphenols inhibit viral infections through different mechanisms. TP = tea polyphenols; 3CL^pro^ = 3-chymotrypsin-like protease; Nrf2 = Nuclear factor erythroid 2 p45-related factor 2. Figure generated with BioRender.

Previous study showed that the 3CL^pro^ structure of SARS-CoV-2 is highly similar to SARS-CoV, which indicates the effectiveness of earlier studies [43]. The key targets of SARS-CoV-2 include three non-structural proteins such as 3CL^pro^, PL^pro^ and RdRp and one structural protein named spike protein (S-protein), which are responsible for replication, transcription, and host cell recognition [50]. In a medicinal plant study evaluating potential inhibition of 3CL^pro^, tea extract has a certain inhibitory effect on 3CL^pro^ of SARS-CoV-2. Inhibition of 3CL^pro^ activity may be able to prevent SARS-CoV-2 replication cycle [51]. Jang et al. cloned the SARS-CoV-2 3CL^pro^ cDNA by chemical synthesis and obtained the SARS-CoV-2 3CL^pro^ protein using a bacterial expression system. It is found that EGCG and theaflavins in tea have a strong inhibitory effect on the 3CL^pro^ of SARS-CoV-2 [52] (Figure 3). The half-maximal inhibitory concentration (IC_50_) of EGCG for SARS 3CL^pro^ is greater than 24 µM, while the IC_50_ of 3CL^pro^ for SARS-CoV-2 is much lower, ranging from 0.847 µM to 16.5 µM. The ECGC IC_50_ values of HCoV-OC43 and HCoV-229E are also higher than SARS-CoV-2 [52]. These results indicate that the inhibitory effect of EGCG on SARS-CoV-2 is more effective than other coronaviruses. In addition to the determination of 3CL^pro^ activity, emerging evidence shows that through molecular docking studies, the binding between polyphenols and 3CL^pro^ is relatively stable and is a potential inhibitor [53,54]. EGCG, ECG, and gallocatechin-3-gallate (GCG) interact with one or two residues of 3CL^pro^. The binding range of the three polyphenols is between −7.6 and −9.0 kcal/mol, among which the affinity for GCG is the lowest and the affinity for EGCG is the highest [53]. Since the 3CL^pro^ of coronavirus is responsible for cleavage of 11 sites of polyprotein, partial inhibition of cleavage by EGCG may help to inhibit coronavirus replication [55]. In general, because 3CL^pro^ plays a key role in virus survival and reproduction, and has no significant homology with known human proteases, its inhibitors have good selectivity and safety expectations. Apart from this, unlike vaccines or monoclonal antibody drugs that cannot prevent variants of S-protein (SARS-CoV-2 mutants emerge one after another and pose a great threat), 3CL^pro^ inhibitors have become a high potential and representative idea for the design of small molecular antiviral drugs, which can be used to design oral antiviral drugs for the treatment of COVID-19 [56].

### 3.3. TP Disrupts the Replication of Coronavirus in Other Ways

The key to preventing SARS-CoV-2 infection is to prevent the binding of the viral S-protein to the cell receptor (ACE2) and inhibit the main protease (3CL^pro^) of the virus. Jang et al. detected the expression level of coronavirus protein with anti-OC43 antibody to observe whether EGCG reduced the production of coronavirus. It was found that EGCG significantly decreased the level of HCoV-OC43 protein in infected cells in a dose-dependent manner. When the concentration of EGCG was 20 mM, the expression of HCoV-OC43 protein was not detected [52].

Nuclear factor erythroid 2 p45-related factor 2 (Nrf2) is an important transcription factor in the defense of cells against oxidative stress, which exists in almost all cells of the human body. When cells are attacked by oxidative stress or exogenous toxic substances, Nrf2 can quickly activate more than 500 genes with cytoprotective function, which can protect cells through antioxidation, detoxification, inhibition of inflammation, promotion of mitochondrial biosynthesis, enhancement of cell autophagy, and so on [57]. Keap1-Nrf2-ARE pathway is the main defense mechanism of antioxidant stress in vivo [58]. Nrf2 activators exert great therapeutic potential against inflammation-related diseases and respiratory diseases by activating this defense pathway. Substances such as EGCG and flavonoids activate Nrf2 and reduce the entry of viruses into host cells. Studies have shown that in the absence of infection, supplementation of EGCG increases the expression of Nrf2-related genes and antiviral mediators and prevents the virus from entering nasal epithelial cells [59]. In vitro and in vivo studies have shown that EGCG stimulates the expression of antioxidant genes in II phase, which is related to Nrf2-EpRE signal transduction [60]. The activation of Nrf2 seems to provide many antiviral effects, which may produce drug resistance to some extent, reduce the rate of viral replication, improve inflammation, and enable people to successfully weather the cytokine storm [61] (Figure 3).

The activation of Nrf2 has been shown to be involved in the response of inflammatory signals to protect lung structure, and the therapeutic effect of Nrf2 has been reported in several animal models of lung diseases, including respiratory infection and ARDS [62]. Nrf2 can inhibit the expression of pro-inflammatory genes such as IL-6 and IL-1β and may also reduce the inflammatory response caused by viral infection by preventing excessive type I IFN [63]. Tests on lung biopsies of patients with COVID-19 showed that the Nrf2 pathway was inhibited [64]. Nrf2 knockout mice showed increased expression of ACE2. Although the exact mechanism of increased expression of ACE2 gene induced by lack of Nrf2 is still unclear, the data show that there is a certain relationship between Nrf2, ACE2 and SARS-CoV-2 [65]. Whether EGCG can activate Nrf2 in vivo to play these possible roles remains to be further studied. The ligands with strong ACE2 binding affinity were screened and it was found that polyphenols were potential candidates.

Most of the potential inhibitors considered to be coronaviruses include conjugated dense ring structures, majority of which are classified as polyphenols [66]. TP has been shown to inhibit influenza A virus RdRp [67]. Dietary resveratrol intake can alleviate the disease of SARS-CoV-2 by regulating the expression and function of ACE2 [68]. TP should be pre-existed in the upper respiratory mucosa at the site of virus infection before it can bind to receptors in vivo. Current studies have shown that the retention of TP in the mucous membranes of the human body after drinking tea may be related to the antiviral effect [69]. The molecular docking study of TP with human ACE2 receptor showed a binding affinity of -8.9 kcal/mol [70]. Although electronic experiments predict an ideal result, more in vitro and in vivo studies are needed to evaluate whether the binding of polyphenols to ACE2 affects virus entry. As EGCG can inhibit the binding of 3CL^pro^ and ACE2 in vitro, whether EGCG can inhibit the replication of coronavirus in vivo remains to be further studied (Figure 3). In summary, these outcomes suggest that EGCG can possibly forestall coronavirus replication [71].

## 4. Immunomodulatory Effects of TP-Mediated Gut-Lung Axis on COVID-19

### 4.1. The Gut-Lung Axis

Lyon reported that pneumonia occurs not only due to lung microbes, but also intestinal microbes seem to have an impact on lung health [72], indicating that there is a close relationship between intestinal flora and lung flora, and alterations in intestinal flora can lead to changes in lung immunity and microecology. A large number of studies have shown that healthy human lungs contain microorganisms, mainly including *Prevotella*, *Streptococcus*, *Veillonella*, *Fusobacterium*, and *Haemophilus* [73,74]. In healthy mice lungs, the microbial biomass is low, comprising 10^3^–10^5^ CFU/g of lung tissue, while in human lungs there are approximately 2.2 × 10^3^ bacterial genomes per cm^2^ [75].

Since the SARS-CoV-2 is a new infectious disease, the prevention and treatment of the SARS-CoV-2 is still in the process of exploration and research. However, for other respiratory tract infection viruses, a large number of studies have confirmed that supplementing with probiotics and prebiotics can significantly reduce the excessive immune response and body inflammation after viral infection, improve immunity, and shorten the course of disease [76]. It has certain reference value for the prevention and treatment of SARS-CoV-2. After prebiotics are fermented by intestinal bacteria, large amounts of short-chain fatty acids (SCFAs), especially butyrate, are produced, which play a significant role in the regulation of the immune system. TP can establish a relatively healthy intestinal environment by preferentially inhibiting pathogenic bacteria in the intestine (having no effect on the growth of beneficial bacteria). Although TP does not meet the definition of standard prebiotics, but in terms of the final effect, it has the same effect as prebiotics [77].

It is worth noting, however, that the gut–lung axis is bi-directional, as many respiratory viral infections are often accompanied by gastrointestinal symptoms [78]. More than 60% of COVID-19 patients have gastrointestinal symptoms such as diarrhea, nausea, and vomiting. Novel coronavirus not only invades the mucosal epithelium of the respiratory tract but may also disturb the intestinal flora and promote intestinal inflammation. This has also been proved in animal models, where intestinal microflora may change in animal models of respiratory tract infection with influenza virus [79] (Figure 2). However, there is a lack of direct evidence of microbial transfer between intestinal microflora and pulmonary microflora, and their role in causing lung or intestinal diseases.

The correlation between the intestinal flora, cytokine levels, and inflammatory markers in patients with COVID-19 suggests that the intestinal flora may participate in the severity of COVID-19 by regulating the host’s immune response [80,81]. In addition, the imbalance of the gut microflora after the disease is resolved may lead to sequelae, which highlights the need to understand how gut microbes are involved in new coronary pneumonia. Similar to the intestinal microbiota, the lung microbiota is considered to play an important role in host immunity by activating the immune system even at low concentrations, and the unbalanced ecosystem in the lungs may be prone to respiratory diseases [82,83].

### 4.2. Immunomodulatory Effects of TP on Microecology

**Figure 4:**
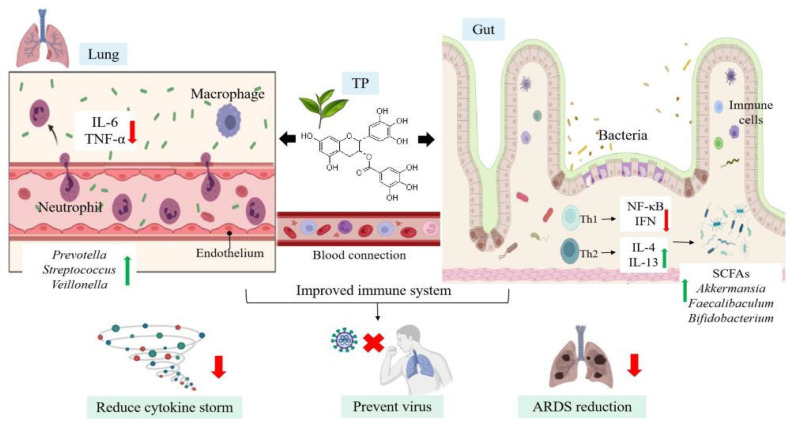
Tea polyphenols increase probiotics in the gut-lung axis, reduce pro-inflammatory factors, and improve the body’s immunity to fight against SARS-CoV-2. Th1 = T helper cell 1; SCFAs = short-chain fatty acids. Figure generated with BioRender.

Immunomodulation includes immune stimulation or immunosuppression of certain cellular and/or humoral immune responses. These defense components prevent pathogens from attaching and entering the host tissue, and once the pathogens are there, mobilize other immune components to resist. Natural products, such as herbs, probiotics, vitamins, polyphenols, and fatty acids, have been shown to have immunomodulatory effects [84]. Many phytochemicals work by targeting TLR and its downstream signaling molecules. TP, especially EGCG and ECG, can significantly enhance the immune response and reduce the risk of inflammation and immune-related diseases [85,86]. As a natural immunomodulator, TP can deal with immune system diseases by up-regulating or down-regulating the immune response without adverse effects such as pro-inflammatory responses [87]. In the human body, T helper cell (Th)1 and Th2 are important members of cellular immunity and humoral immunity, respectively. The dynamic balance of Th1/Th2 affects immune function. TP blocked the upregulation of NF-κB inhibited the transcription and secretion of pro-inflammatory cytokine IFN-γ secreted by Th1 cells [88], and increased the level of anti-inflammatory cytokine IL-4 necessary for Th2 cell proliferation and activation [89] (Figure 4).

Oral administration of EGCG instead of rectal infusion reduced dextran sulfate sodium (DSS)-induced colitis and enhanced the integrity of the colon barrier in mice. By increasing Akkermansia abundance and butyrate production, the authors observed significant EGCG-mediated changes in the intestinal microbiome. In DSS-induced mice, EGCG intake significantly enriched SCFAs and produced more beneficial bacteria, such as Akkermansia, Faecalibaculum, and Bifidobacterium. In addition, prophylactic administration of EGCG could reduce the levels of plasma IL-1β, IL-6, IL-8, and TNF-α in mice with colitis induced by DSS, indicating that EGCG can alleviate the symptoms of colitis, colonic injury, and inflammation [77].

The stable and elastic symbiotic relationship between intestinal immune system and microflora affects host health [90]. Intestinal mucosal immune system is one of the components of intestinal microecology. Intestinal intraepithelial lymphocytes and lamina propria lymphocytes are the main immune cells of intestinal tract. Dietary supplementation of TP effectively prevented the increase of pro-inflammatory cytokines in serum and liver of mice induced by high-fat diet. The changes of secretory immunoglobulin A in the small intestine, serum, and feces further proved the improvement of intestinal mucosal immunity in mice treated with EGCG [91].

### 4.3. Healthy Microecology Enhance Immune Response to Fight against SARS-CoV-2

Although respiratory tract is the chief site of SARS-CoV-2 infection and respiratory transmission is the main route of SARS-CoV-2 infection, recent studies have shown that SARS-CoV-2 can also infect the gastrointestinal tract, and fecal-oral transmission may be another route of coronavirus transmission [92,93,94]. The intestinal immune system is one of the most extensive networks of immune cells in the human body. Intestinal immune cells participate in the regulation of intestinal flora and enhance the function of intestinal epithelial barrier. Intestinal mucosal symbiotic bacteria play an important role in intestinal homeostasis, which can not only provide nutrition and produce important metabolites for the body, but also promote the maturity of the immune system [95]. Dietary is one of the main factors affecting the diversity and function of intestinal flora. A nutritionally balanced diet rich in dietary fiber can maintain a healthy intestinal flora [96].

Strengthening the host immune system is an important factor in the fight against viral infection. Virus infection disrupts the normal interaction of the microbial community [36]. The imbalance of intestinal flora will lead to the destruction of the epithelial barrier and the lack of immune response of intestinal flora, resulting in chronic inflammation and tissue damage [97]. One of the main causes of SARS-CoV-2 death is cytokine storm caused by overactivation of the human immune system. Cytokine storm is characterized by a sudden and sharp increase in the levels of different proinflammatory cytokines in the circulation of the body [98]. Some people infected with SARS-CoV-2 can cause overactive and uncontrolled immune response. In addition to antiviral therapy, anti-inflammatory therapy that suppresses cytokine storm is essential to reduce mortality in patients with COVID-19. IL-6 is often elevated in patients with COVID-19, and its level is positively correlated with COVID-19 mortality [87,99]. Persistent cytokine storm increases the alveolar vascular permeability, the body fluid and blood cells enter the alveoli, resulting in pulmonary edema, ARDS, and even respiratory failure [100,101]. Once a cytokine storm occurs, the immune system kills not only the virus, but also a large number of normal cells in the lungs. Tissues and organs with high expression of ACE2 may become the main targets of SARS-CoV-2 invasion and turn into the battlefield between SARS-CoV-2 and immune cells, resulting in multiple organ failure and life-threatening [102]. Low to moderate doses of EGCG showed inhibitory effects on acute lung injury and ARDS in rodent models, including RNA virus [103]. If these activities can be confirmed in humans, EGCG may help contain cytokine storms and ARDS caused by SARS-CoV-2 (Figure 4). Therefore, the reconstruction of immune homeostasis through the normalization of intestinal microflora is considered to be a valuable method for the treatment of human diseases and infections.

## 5. Conclusions and Future Perspectives

It has been found that using TP to target viral proteins or block cell receptors is a seemingly reasonable antiviral method. As the security of tea has long been demonstrated, a suitable amount of TP admission can be straightforwardly utilized for in vivo experiments without toxic problems. At present, most of the data of EGCG come from in vitro studies, so more animal or clinical trials are expected to affirm the effect of EGCG on SARS-CoV-2. Polyphenols can enhance the body’s anti-inflammatory and antioxidant defense against viral infection and enhance the body’s immunity by increasing probiotics. No disease should be treated alone because organs interact with each other through various networks of connections. Understanding the crosstalk mechanism between intestinal and lung defense and the role of intestinal and pulmonary microflora in regulating and maintaining immune system homeostasis is a promising research field of anti-novel coronavirus. As TP is easy to be oxidized or transformed into other structural forms before entering the target, more research on the change mechanism of TP in vivo is needed to ensure that it can be developed into an effective antiviral drug or supplement in the future.
